# Revising the Taxonomic Distribution, Origin and Evolution of Ribosome Inactivating Protein Genes

**DOI:** 10.1371/journal.pone.0072825

**Published:** 2013-09-05

**Authors:** Walter J. Lapadula, María Virginia Sánchez Puerta, Maximiliano Juri Ayub

**Affiliations:** 1 Área de Biología Molecular, Departamento de Bioquímica y Ciencias Biológicas, UNSL and Instituto Multidisciplinario de Investigaciones Biológicas de San Luis (IMIBIO-SL-CONICET), San Luis, Argentina; 2 Instituto de Ciencias Básicas, IBAM-CONICET and Facultad de Ciencias Agrarias, Universidad Nacional de Cuyo, Mendoza, Argentina; American University in Cairo, Egypt

## Abstract

Ribosome inactivating proteins are enzymes that depurinate a specific adenine residue in the alpha-sarcin-ricin loop of the large ribosomal RNA, being ricin and Shiga toxins the most renowned examples. They are widely distributed in plants and their presence has also been confirmed in a few bacterial species. According to this taxonomic distribution, the current model about the origin and evolution of RIP genes postulates that an ancestral RIP domain was originated in flowering plants, and later acquired by some bacteria via horizontal gene transfer. Here, we unequivocally detected the presence of RIP genes in fungi and metazoa. These findings, along with sequence and phylogenetic analyses, led us to propose an alternative, more parsimonious, hypothesis about the origin and evolutionary history of the RIP domain, where several paralogous RIP genes were already present before the three domains of life evolved. This model is in agreement with the current idea of the Last Universal Common Ancestor (LUCA) as a complex, genetically redundant organism. Differential loss of paralogous genes in descendants of LUCA, rather than multiple horizontal gene transfer events, could account for the complex pattern of RIP genes across extant species, as it has been observed for other genes.

## Introduction

Ribosome inactivating proteins (RIPs; EC 3.2.2.22) irreversibly modify ribosomes through its RNA *N*-glycosidase activity that depurinates an adenine residue in the conserved alpha-sarcin/ricin loop (SRL) of 28S rRNA [1]–[4]. Such modification prevents the binding of elongation factor 2 (EF-2) to the SRL, arresting protein synthesis [5], [6].

Even though several RIPs have been extensively studied at the biochemical level, their biological role(s) remains open to speculation. In some cases, it seems reasonable to predict their functions. For instance, the high toxicity of ricin supports an antifeedant role for this protein, whereas shiga and shiga-like toxins are strong virulence factors for their harboring bacteria. Antiviral and other defense activities were postulated for other RIPs, but no definite evidence has been obtained.

Classically, RIPs have been classified as type 1 and 2. Type 1 RIPs are single-domain (PF00161) proteins found in many plant and a few bacteria species, whereas type 2 RIPs are two-domain polypeptides. In the latter proteins, the RIP domain (A-chain) is fused to a C-terminal lectin domain (B-chain) (PF00652). Type 2 RIPs have been found exclusively in plants, leading to the hypothesis that fusion of RIP and lectin domains took place once in the flowering plant lineage [7]. More recently, a third class of RIPs (named type 3 RIPs) has been described, in which the RIP domain is fused to a C-terminal domain with no obvious similarity to any protein of known function [8], [9]. These type 3 RIPs were only found in the Poaceae and their C-terminal domain was named C-chain. Based on these observations, a new nomenclature for RIPs was proposed, in which they are termed **A** (type 1), **AB** (type 2) or **AC** (type 3) **RIPs** [7]. In the present work, we use this naming scheme.

Several RIPs (e.g. ricin, shiga-like toxins, trichosanthin) were deeply characterized at the biochemical and molecular level. In contrast, the molecular evolution of RIP genes was discussed only in a few research and review papers [7], [9]–[12]. The currently accepted hypothesis about the origin and evolution of RIP genes postulates that an ancestral RIP domain was originated in flowering plants, and later acquired by some bacteria via horizontal gene transfer (HGT) [7]. This model is supported by the relatively wide distribution of the RIP domain in plants, its scarce presence in bacterial genomes, and the absence of reported RIP domains in other lineages; i.e. fungi, metazoa or archaea. A drawback of this model is that RIP genes are present in gram-positive and gram-negative bacteria [7], [10], which diverged earlier than the appearance of plants. Thus, the current model requires at least two independent HGT events from plants to bacteria. As indicated by Glansdorff *et al*. [13], [14], the proposal of HGT events should be taken with caution when a simpler mechanism, such as differential loss of paralogous genes, is sufficient to explain the observed data.

The goals of the present work are: i) to gather new information about the origin and the evolutionary history of RIP domains, and ii) to evaluate whether the new data support or not the current model involving HGT. We used a bioinformatic approach to identify novel RIP genes in all domains of life. Notably, we found clear evidence for the presence of RIP domains in Fungi and Metazoa. All RIP sequences were analyzed to infer their phylogenetic history. Based on all these new data, we propose an alternative hypothesis about the origin and evolution of the RIP domain, in which it is not necessary to postulate HGT events. Advantages and potential drawbacks of this new hypothesis are discussed.

## Materials and Methods

### Data mining and search for novel RIPs genes

**Figure 1:**
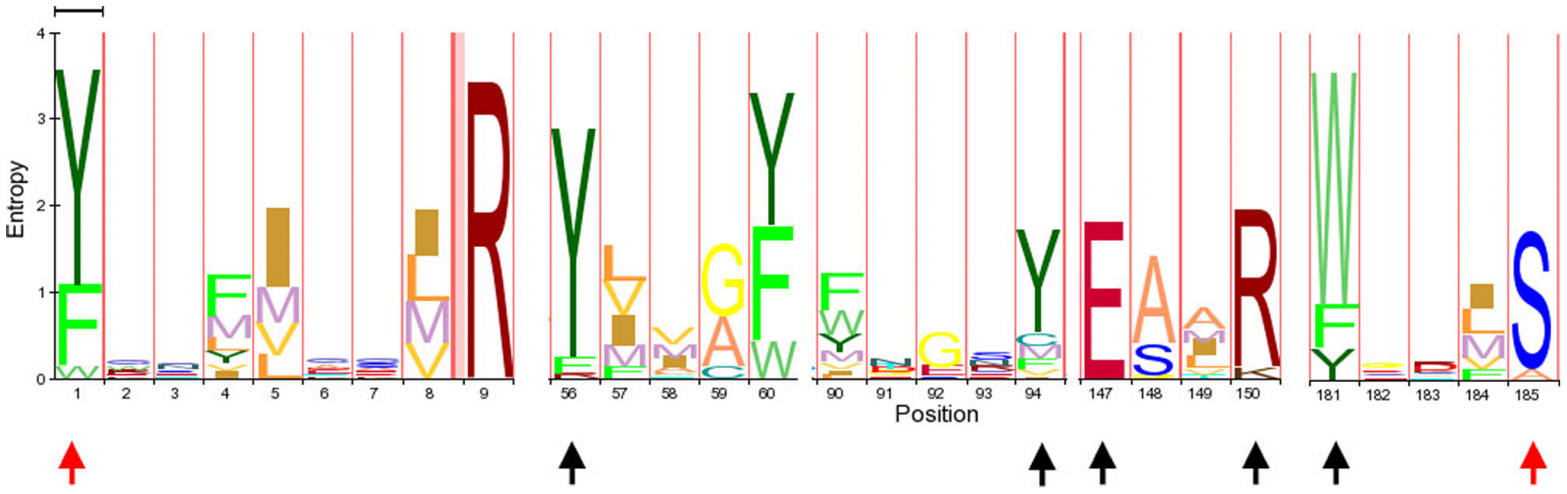
Logo representation of the sequence alignment used for HMMER search. Residues forming the active site are indicated by black arrows. Conserved residues used to define the conserved region of the RIP domain, as previously described [10], are indicated by red arrows.

We used the amino acid sequences of previously reported RIPs [10] as queries for BLASTP and TBLASTN searches (http://blast.ncbi.nlm.nih.gov/Blast.cgi) against different protein (nr) and nucleotide (WGS, ESTs, nr, RefRNAseq) databases under default parameters. Retrieved sequences were curated by confirming that each sequence belonged to the RIP superfamily domain (PF00161) using Pfam (http://pfam.sanger.ac.uk/) and checking the presence of the amino acids predicted to form the active site. Eighty eight representative protein sequences were aligned using MAFFT [15] (http://mafft.cbrc.jp/alignment/software/) under default parameters (Dataset S1). This matrix (Figure 1) was used to perform new searches on protein databases using the HMMR search [16] tool under default conditions (http://hmmer.janelia.org/).

### Phylogenetic analysis

We selected 100 representative RIP amino acid sequences to perform phylogenetic analyses using Bayesian Inference (MB) and Maximum Likelihood (ML). For this, a multiple amino acid sequence alignment was constructed based on the conserved region shown in Figure 1. The alignment (Dataset S2) was obtained using T-COFFEE [17], [18] (http://tcoffee.vital-it.ch/) under default parameters. Similar results were obtained using other alignment algorithms, such as MAFTT [15] and ClustalW [19] (data not shown). The WAG substitution matrix and gamma distribution model with invariable sites was selected as the model that best fits our data set using ProtTest [20]. PhyML was run using the algorithm Tree-Bisection-Reconnection (TBR) [21] with 5 initial starting random trees. To estimate the robustness of the phylogenetic inference, we ran 100 bootstrap (BS) replicates. Bayesian inference using Mr. Bayes 3.1.2 [22] was run for 2×10^6^ generations and the average standard deviation of split frequencies was <0.01. Finally we constructed a consensus tree from ML and MB trees.

## Results and Discussion

### RIP genes are present in fungal and metazoan genomes

To date, the presence of RIP genes has only been confirmed in plants and a few bacteria [7]. However, indirect evidence suggested the presence of translation inhibitory activity compatible with the presence of RIPs in a few fungi such as *Volvariella volvacea* [23], *Flammulina velutipes* [24], [25] *, Lyophyllum shimeji* [26], *Hypsizigus marmoreus* [27] and *Pleurotus tuber-regium* [25]. Unfortunately, no genomic data are available for these fungal species. Moreover, only in one case (*Volvariella volvaceae*) depurinating activity was demonstrated [23]. Since translation inhibitory proteins other than RNA N-glycosidases have been described in fungi (i.e. sarcin; a rRNA endonuclease, EC 3.1.27.10), it is not possible to conclude that RIP genes are actually present in the genomes of these species. Similar evidence is available for metazoa. It has been reported that extracts of some mammalian tissues had adenine glycosidase activity compatible with RIP activity. However, no protein synthesis inhibition by these extracts could be demonstrated [28], and no sequences with significant similarity to RIPs have been reported.

In order to shed new light on the possible existence of RIP genes in organisms other than plants and bacteria, we performed exhaustive and iterative TBLASTN searches on different nucleotide database (WGS, ESTs, Nr, RefRNAseq), as well as BLASTP searches on protein databases, using a previously reported set of sequences [10] as queries. Notably, we found nine fungal sequences (Table S1) with low but significant amino acid sequence similarity (E-values ranged from 1×10^−37^ to 6×10^−14^; identity values ranged from 27% to 39%) when searching with at least one of the query sequences. Analysis of these sequences revealed that all five residues predicted to form the active site were conserved. In addition, we confirmed that these sequences encoded a canonical RIP domain (PF00161) using Pfam, as described in Materials and Methods. No hits belonged to Metazoa. Moreover, searches restricted to plant and bacterial databases using the novel fungal sequences as the query, revealed the presence of additional, previously non-reported RIP genes in these taxa (Table S1).

**Figure 2:**
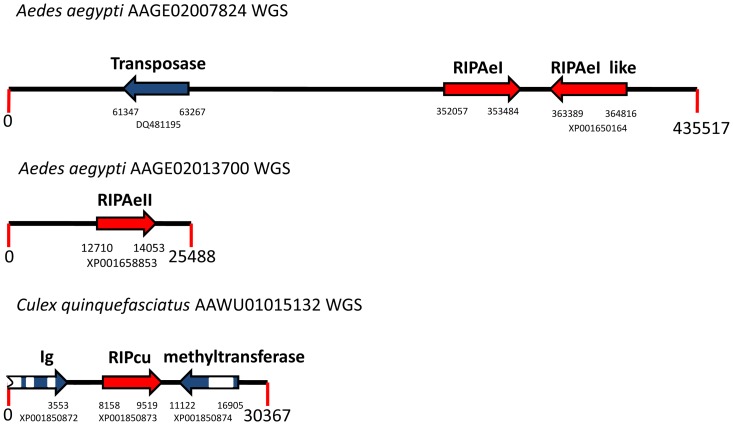
Schematic representation of metazoan RIP genes (red arrows) and their neighbor genes (blue arrows) from *Aedes aegypti* and *Culex quinquefasciatus*. Accession codes for protein sequences and the nucleotide position for the first and the last nucleotide of the ORF are shown below each arrow. Introns are indicated as white boxes. GenBank accession numbers for each WGS sequence are indicated next to the taxon name.

**Figure 3:**
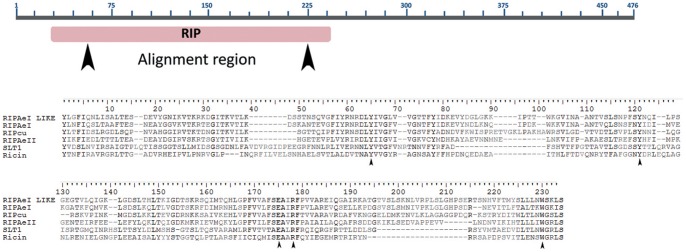
Sequence analysis of metazoan RIPs. **A.** Schematic representation of the metazoan RIP AeI-like sequence. The predicted protein harbors 476 amino acids. The RIP domain region is shown in red. Arrowheads indicate the conserved region used for sequence alignment in B. **B.** Sequence alignment of the conserved RIP region from metazoan RIPs along with SLT-1 and ricin. Arrowheads indicate those residues predicted to form the active site.

These results strongly suggested that BLAST searches would not allow finding all RIP genes, because of the high sequence divergence among some of these sequences. Therefore, we took a different, complementary, approach to find novel RIP domains in the sequence databases. It has been shown that sequence comparison using multiple sequence alignment profiles are more efficient than pairwise methods to detect remote homologues [29]. Therefore, we performed searches on protein databases, using HMMER (http://hmmer.janelia.org/) as described in Materials and Methods. By using this approach, we were able to detect a novel set of sequences encoding RIPs in plants and bacteria (Table S1). Most importantly, we found, for the first time, four RIP-encoding sequences in metazoa. Three paralogous genes were found in two WGS accessions from *Aedes aegypti* (AAGE02007824, AAGE02013700), and a fourth gene was present in a WGS accession from *Culex quinquefasciatus* (AAWV01015132). Both species are members of the Culicinae family. All these sequences are expressed at the mRNA level, given that we identified their corresponding ESTs (FF167149, DV375198, EE993988, DV312220, respectively). To rule out any artifact on these WGS sequences (e.g. DNA contamination), we analyzed the genomic context of the new RIP genes to identify other genes in their neighborhood (Figure 2). Predicted proteins encoded by these neighboring genes were analyzed by TBLASTN searches, and the highest score matching sequences belonged to insects. Next, we analyzed the amino acid sequence of the metazoan RIPs. With the exception of RIPAeII protein, all sequences were recognized by Pfam as RIPs. Moreover, a sequence alignment of metazoan RIPs along with the canonical RIPs, ricin and SLT-1, showed that the five residues predicted to form the active site of RIPs were conserved (Figure 3). These results strongly suggested that these sequences were genuine RIP genes from Metazoa, ruling out artifacts in the databases.

### Alternative hypothesis for the origin and evolution of RIP genes

**Figure 4:**
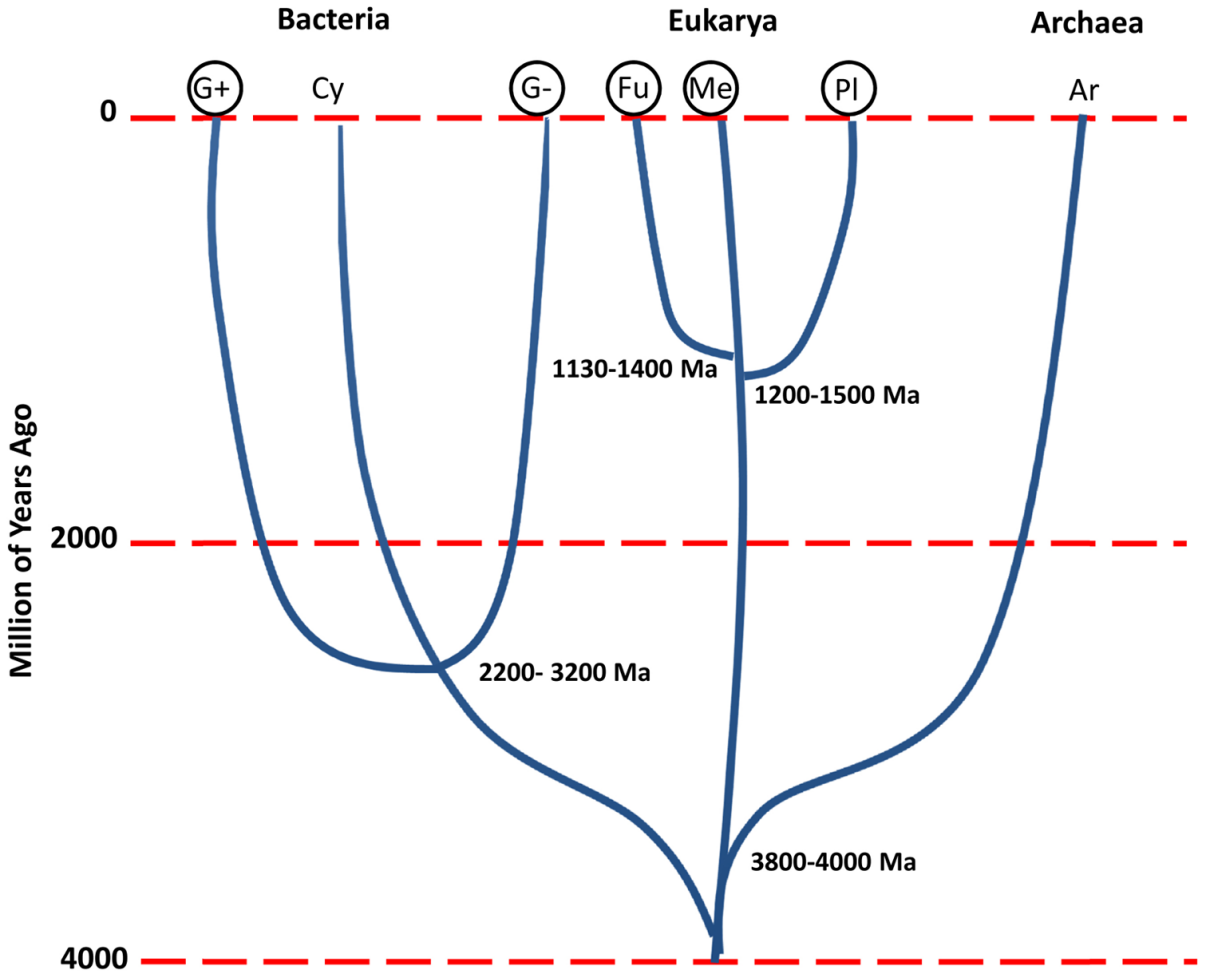
Schematic representation of the estimated divergence times among the main lineages of life. Gram positive (G+), Gram negative (G−) and Cyanobacteria (Cy) diverged 2200–3200 Mya. Plant (Pl), Fungi (Fu), Metazoa (Me) separated approximately 1130–1500 Mya, being the lineage leading to plants the first to diverge. [30]–[32]. Circles around the taxon name indicate those lineages where the presence of RIPs genes has been demonstrated.

Our findings substantially altered the current understanding of RIP gene distribution across life. We presented clear evidence that RIP genes are present in organisms other than plants and bacteria. In addition, we found several new bacterial and plant RIP genes (Table S1). In some cases, they belonged to genera of bacteria where no RIP-encoding sequence had been previously reported, such as *Flavobacterium* and *Corynebacterium*. The occurrence of RIP domains in fungi and metazoa, along with a wider (than previously recognized) distribution in bacteria, strongly challenges the current hypothesis of RIP domain being originated in flowering plants. The main weaknesses of the current model [7] include the following:

i) At least two independent HGT events are required to explain the wide distribution of RIPs in bacteria. RIPs are present in both gram-positive and gram-negative bacteria, which diverged about 2200–3200 millions of years ago (Mya), while the origin of plants took place 1200–1500 Mya (Figure 4) [30], [31];

ii) Given that the plant lineage diverged earlier than fungi and metazoa during evolution (Figure 4) [32], if RIP genes were originated in plants, at least one additional independent HGT is needed to explain the presence of these genes in fungi and metazoa.

These additional HGT events required under the current model of RIP evolution to explain the new data led us to propose an alternate, more parsimonious, model of RIP gene origin and evolution. We postulate that RIP genes were originated much earlier during evolution. In fact, according to our hypothesis, several of these genes were already present in the common ancestor of bacteria and eukaryotes. This model fits better with the presence of RIP genes in diverging organisms such as gram-positive and gram-negative bacteria, fungi, metazoa and plants. The main drawback of this new hypothesis is our current inability to find RIP genes in the third domain of life, the Archaea. This could be due to poor genomic coverage and/or annotation (HMM searches are restricted to annotated proteins) of these organisms or due to limitations of the search algorithms. Taking into account that the presence of RIP genes in fungi and metazoa remained unknown until now, it is reasonable to think that future, improved data mining strategies will allow us to identify RIP genes in other lineages too. For instance, during the review process of this manuscript, we found, for the first time, a RIP encoding sequence that belongs to cyanobacteria (protein id YP_007137128). In addition, the presence of introns (highly frequent in metazoan genomes) may be responsible, at least partially, for a decreased detection rate of these genes. On the other hand, we cannot rule out the possibility that archaeal genomes are actually devoid of RIP genes, due to a gene loss event in the cenancestor of these organisms. It is expected that gene loss would be much more likely than gene acquisition via HGT. Next, we show evidence that loss of RIP genes is a very common process during evolution, even in plants were they are relatively more abundant.

### Loss of RIP encoding sequences is a frequent event in evolution

**Figure 5:**
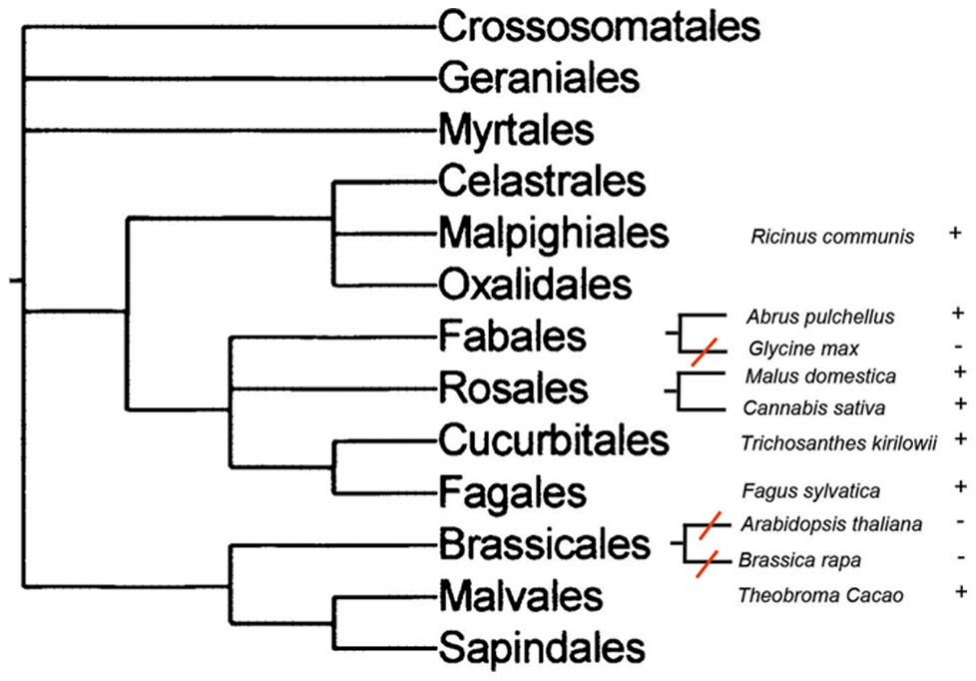
Schematic representation of phylogenetic relationships among several plant lineages of rosids, taken from a previous report [41]. Species are indicated with (+) and (−), according to the presence or absence of RIPs genes, respectively. Red lines represent the inferred RIP gene loss events.

We analyzed the distribution of RIP genes in different plant taxa. Loss of RIP genes can be demonstrated by the absence of RIP genes in a species embedded in a RIP-containing clade. This would imply that RIP genes were present in the common ancestor of these lineages and were lost in one of them. Therefore, we searched for plant species whose genomes have been fully sequenced and no RIP genes were detected by similarity searches. Then, we searched for RIP genes in closely related species. Figure 5 shows a schematic representation of our findings. RIP genes are present in Fabales (*Abrus pulchellus*), Rosales (*Malus domestica*, *Cannabis sativa*), Cucurbitales (*Trichosanthes kirilowii*) and Fagales (*Fagus sylvatica*). On the other hand, RIP genes cannot be found in *Glycine max*, a species that is closely related to *Abrus pulchellus*. This indicates a RIP gene loss event in the lineage leading to *Glycine max*, after its divergence from *Abrus*. Another event of RIP gene loss can be inferred in the order Brassicales, since *Arabidopsis thaliana* and *Brassica rapa* lack RIP genes, whereas three RIP genes are present in *Theobroma cacao*, belonging to the Malvales order (Figure 5). Finally, the absence of AB RIPs in *Oryza sativa*, which is closely related to other Poaceae harboring a set of closely related AB RIPs such as *Sorghum* (XM002459548), *Saccharum* (CA078531), *Zea* (AY105813) and *Phyllostachys* (FP092597) strongly suggests another case of gene loss, even when a deletion of B-chain gene cannot be ruled out.

### Phylogenetic analyses support the hypothesis of several paralogous RIPs in the common ancestor of bacteria and eukaryotes

**Figure 6:**
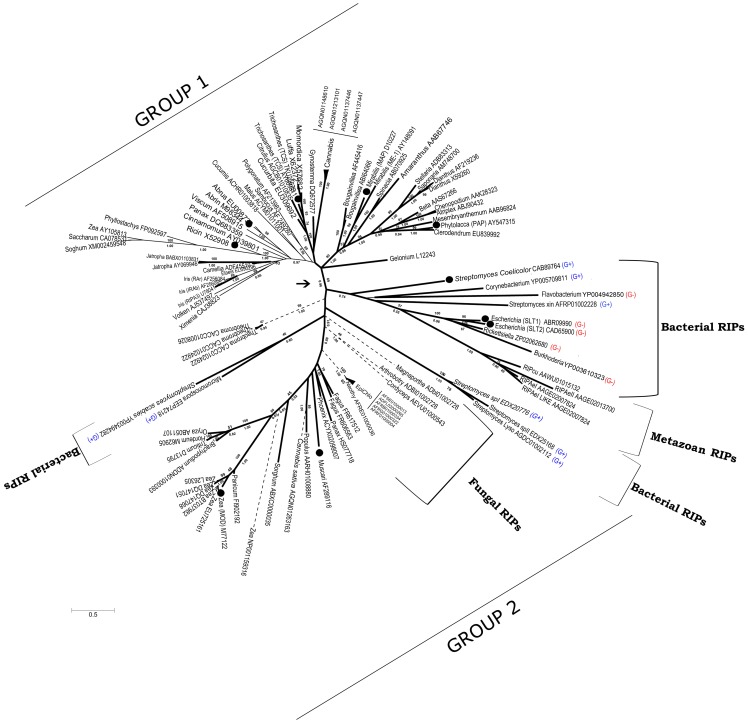
Consensus phylogenetic tree of RIPs based on ML and MB analyses. Numbers below branches indicate Bayesian Posterior Probabilities (BPP) and numbers above branches are Bootstrap Support (BS) values from the ML analysis. The arrow indicates the node separating Group 1 and Group 2 of RIP genes (see the text for details). A, AB, and AC RIPs are indicated by thick continuous, thin continuous, and dashed lines, respectively. Black circles indicate those RIPs with demonstrated RNA N-glycosidase activity. GenBank accession numbers are shown for each sequence. G+ and G- indicate Gram positive and Gram negative bacteria, respectively.

Our discovery of RIP genes in fungal and metazoan genomes challenges the hypothesis of RIP genes originating in flowering plants. To test this hypothesis further, we performed phylogenetic analyses of available RIP sequences (Figure 6). The phylogeny of RIP genes was incongruent with the phylogenetic relationships among the organisms containing those genes. One of the most clear examples is the strong relationship between AB RIPs from the monocot *Polygonatum multiflorum* (AF213983) and the dicot *Sambucus nigra* (AF249280), supported by high boostrap (BS: 89) and Bayesian Posterior Probability (BPP: 0.99) values (Figure 6; [33]). These discrepancies between the RIP gene tree with the species tree are compatible with multiple HGT events and/or the existence of multiple ancestral paralogous genes followed by lineage-specific gene loss.

Figure 6 shows two separate groups of RIP sequences supported by BS and BPP values of 69 and 0.96, respectively. Group 1 contains all AB RIPs and most of the plant A RIPs that have been biochemically characterized. On the other hand, Group 2 contains RIPs from bacteria, fungi, plants and metazoa. This group includes some RIPs which have been biochemically characterized such as shiga and shiga-like toxins [34], a RIP from *Streptomyces coelicolor* [35], Musarmin from *Muscari armeniacum* [36] and one type A RIP from maize [37].

Another interesting observation is that highly divergent sequences are found in gram-positive and gram-negative bacteria, a fact that is very difficult to explain by a rather recent HGT from plants. Moreover, several paralogous genes with very low amino acid identity (around 20%) are present within the genus *Streptomyces*, strongly suggesting that these genes diverged very early in evolution.

### Origin of AB and AC RIPs

AB RIPs have been found exclusively in plants, suggesting that the fusion of A and B domains took place once in the flowering plant lineage [7], followed by the deletion of the B-chain in several secondary A RIPs. Our phylogenetic analyses support this conclusion, because AB RIPs (thin continuous lines in Figure 6) form a monophyletic group, taking into account those secondary A RIPs.

On the other hand, AC RIPs have been reported only for Poaceae, leading to the hypothesis that fusion between A and C domains took place in this lineage [7]. Interestingly, we found one AC RIP in the dicot *Cannabis sativa* and several AC genes in fungi (dashed lines in Figure 6). All these sequences displayed significant similarity (E-value ranging from 9×10^−6^ to 1×10^−15^ using BLASTP) to the C domain of JIP60; the prototypical AC gene. Therefore, it seems likely that the A–C fusion occured before plants and fungi diverged. Finally, it is interesting to note that at least in one Poaceae species (*Zea mays*), all three classes of RIPs; namely A, AB and AC are present (GenBank Accessions M77122, AY105813 and NP001159316, respectively). This observation further supports the hypothesis of multiple paralogous RIP genes, and lineage-specific gene losses.

The present data show that at least two different A-chain paralogous genes were independently fused to B and C domains, leading to the current AB and AC RIP genes, respectively.

## Conclusions

In summary, data from this study, along with previous information, prompted us to propose a more parsimonious model on the origin and evolution of the RIP domain. The emerging picture can be summarized as follows:

i) Initially, the RIP domain was present in the common ancestor of bacteria, archaea and eukaryotes. Taking into account the largely diverging sequences found in *Streptomyces* spp (distant paralogs), we propose that several paralogous RIP genes had already evolved before the three domains of life diverged.
ii) After the divergence of different lineages, multiple gene duplication and gene loss events of paralogous genes took place, yielding a high heterogeneity in the number of RIP genes among organisms.
iii) After the plant lineage diverged, at least one of these paralogous genes suffered multiple duplications, giving origin to the great diversity of plant RIPs. This was probably due to the acquisition of novel functional roles. In addition, the frequent polyploidization event in plants could have impacted on the multiplication of RIP genes.
iv) As previously proposed [7], one plant paralogous RIP domain fused to a lectin domain, giving rise to AB RIPs.
v) Also, according to Peumans and Van Damme's model [7], some AB RIP genes suffered a deletion of the lectin domain originating “secondary” A RIPs. A clear example is the A RIP from *Iris hollandica* [38], which is closely related to AB RIPs from the same species with high support (BS: 89, BPP: 1) (Figure 6).
vi) Before the divergence of fungi and plants, a paralogous RIP gene fused to a C-chain domain, originating AC RIPs, which are present in several monocots, at least one dicot (*Cannabis sativa*) and fungi.

Our model about RIP genes' origin and evolution is in line with the current conception of LUCA as a complex, genetically redundant organism. Differential loss of paralogous genes in the descendants of LUCA could account for the complex pattern of RIP genes across extant species, as it has been demonstrated for other genes [39], [40].

## Supporting Information

Dataset S1 **Data matrix.** The aligned sequence data is presented in Fasta format.
(FAS)

Dataset S2 **Data matrix.** The aligned sequence data is presented in Fasta format.
(FAS)

Table S1 **Summary of RIPs genes from plants, bacteria, fungi and metazoan used in the present work which have not been previously reported** **[7], [10]** **.** The first column indicates the RIP name used for identification in Figure 6. The second column indicates the organism harboring each gene. The third column indicates the Genbank code access or Protein ID.
(DOC)
