# Supplementary material for: Revising the Taxonomic Distribution, Origin and Evolution of Ribosome Inactivating Protein Genes
Source: PLoS One. 2013 Sep 5;8(9):e72825. doi: 10.1371/journal.pone.0072825 (PMC3764214; doi:10.1371/journal.pone.0072825)
Supplement: Table S1 — Summary of RIPs genes from plants, bacteria, fungi and metazoan used in the present work which have not been previously reported [7], [10] . The first column indicates the RIP name used for identification in Figure 6. The second column indicates the organism harboring each gene. The third column indicates the Genbank code access or Protein ID. (DOC) [file pone.0072825.s003.doc]

| **Bacterial RIPs** | Organism | GenBank or (Protein ID) |
| --- | --- | --- |
| Burkholderia | *Burkholderia sp* | (ZP06230219) |
| Corynebacterium | *Corynebacterium ulcerans* | (YP005709811) |
| Flavobacterium | *Flavobacterium columnare* | (YP004942850) |
| Streptomyces lyso | *Streptomyces lysosuperificus* | AGDC01002112 |
| Streptomyces spI | *Streptomyces sp* | (EDX20776) |
| Streptomyces spII | *Streptomyces sp* | (EDX25168) |
| Streptomyces xin | *Streptomyces xinghaiensis* | AFRP01002228 |
|  |  | |
| **Fungal RIPs** |
| Arthrobotry | *Arthrobotrys oligospora* | ADOT01000134 |
| Cordyceps | *Cordyceps militaris* | AEVU01000543 |
| EpiChloI | *Epichloe glyceriae* | AFRF01000004 |
| EpiChloII | *Epichloe amarillans* | AFRB01000334 |
| EpiChloIII | *Epichloe typhina* | ADFL02000265 |
| EpiChloIV | *Epichloe festucae* | AFRG01000322 |
| EpiChloV | *Epichloe brachyelytri* | AFSE01000013 |
| Magnaporthe | *Magnaporthe poae* | ADBL01002728 |
| Neothy | *Neotyphodium gansuense* | AFRE01000036 |
|  |  | |
| **Metazoa RIPs** |
| RIPAeI | *Aedes aegypti* | AAGE02007824 |
| RIPAeI LIKE | *Aedes aegypti* | AAGE02007824 |
| RIPAeII | *Aedes aegypti* | AAGE02013700 |
| RIPcu | *Culex quinquefasciatus* | AWU01015132 |
|  |  | |
| **Plant RIPs** |
| Amaranthus | *Amaranthus tricolor* | (AAB67746) |
| Atriplex | *Atriplex patens* | (ABJ90432) |
| Brachypodium | *Brachypodium distachyon* | ADDN01000393 |
| Cannabis | *Cannabis sativa* | AGQN01263163 |
|  |  | AGQN01148610 |
|  |  | AGQN01213101 |
|  |  | AGQN01137446 |
|  |  | AGQN01137447 |
| Citrullus | *Citrullus lanatus* | AGCB01010835 |
| Chenopudium | *Chenopodium album* | AAK28323 |
| Cucumis | *Cucumis sativus* | ACHR01003818 |
| Fagus | *Fagus Sylvatica* | FR617512 |
|  |  | FR606563 |
| Panax | *Panax ginseng* | HS077718 |
| Phoenix | *Phoenix dactylifera* | ACYX02098007 |
| Stellaria | *Stellaria media* | (ADB83313) |
| Sorghum | *Sorghum bicolor* | ABXC01000035 |
| Theobroma | *Theobroma cacao* | CACC01024922 |
|  |  | CACC0102492 |
|  |  | CACC01008026 |
| Ximenia | *Ximenia americana* | (CAJ38823) |

**Supplementary Table 1:** Summary of RIPs genes from plants, bacteria, fungi and metazoan used in the present work which have not been previously reported [7, 10]. The first column indicates the RIP name used for identification in Figure 6. The second column indicates the organism harboring each gene. The third column indicates the Genbank code access or Protein ID.
